# National Board of Health

**Published:** 1880-03

**Authors:** 


### NATIONAL BOARD OF HEALTH.

Ostensibly to promote public sanitation, the National Board of Health asks of Congress additional powers. People of the States are jealous of their rights as such, and any infringement upon them by the general government is resisted by all justifiable means.

The additional powers sought, will give the Board complete control of river and railroad transportation and travel, as well as municipal and State sanitation. This gigantic fraud, under the name of the National Board of Health, will take care of us by a Federal police system, to which obedience is to be enforced by the strong arm of the government.

Well, if the right to travel unmolested in the interior of a State and to ship goods on railroads through the same, be taken away, we have no further use for State lines, nor the farce of municipal and State governments. If our representatives (?) in Congress give these rights away, we should by one general demonstration decline such representation. Medical men are not more interested personally in this matter than other citizens, but they know well that the ostensible object of quarantinists is a consummate fraud. It is true that we are met at this point by the fact that the movement was commenced by a medical man in the profession, and sanctioned by all the medical men called to assistant in its operation. We will not say that all medical men believe its objects unattainable and the pretentions of its advocates a fraud on the public, but we do say that the prestige of government medical officers, the distribution of half a million of money, the pressure made upon Congress by interested medical men and the general pressure and influence exerted throughout the country, have conspired to make quarantine against yellow fever appear reasonable, and silence those whose judgment disproved the course.

Thus it is that physicians who do not believe the fever can be communicated from one person to another, and consequently opposed to quarantine for protection, dislike to oppose high authority, and what seems to be the expressed public opinion. Such fall into a kind of compromise and say, "the proper circumstances must exist," and that the disease may "be contracted from local cause and from importation." To such milk-and-cider confessions, instead of a square out defense of the truth, is attributable the trouble now being brought upon the country. When I say small-pox is contagious, I cannot object to a law compelling vaccination, and if I admit that yellow fever comes by cases being imported and transported, I could not reasonably object to coast and inland quarantine. No such protection against the fever, as that afforded by vacination against the small-pox has been discovered, and if yellow fever be contagious, of course quarantine against those who have the disease is the only means of protection.

But for the urgent and noisy demand of interested physicians, and the silence of those who do not agree with them, legislators would, with the history of the fever in Memphis last year before them, decide that quarantine is entirely unnecessary for protection. Instead of ordering the ruinous quarantine system proposed, they would pass a bill ordering their appropriations expended in draining and otherwise removing the stagnant pools, marshes and bayous around Memphis and other places before the warm season. They would order quarantine against the floods coming down the river instead of the people and merchandise from below. We heartily endorse and concur in the protest against the proposed additional quarantine powers, but must be allowed to make the confession that our reticence and want of moral courage to oppose the errors of those in official position and high authority, has in a good degree been the means of bringing this trouble upon the country, and the danger of losing the dearest rights of the people.

The people of Savannah seem to be alive on this subject. That which is sought to be enacted, ostensibly, for their benefit and safety, they know, will be highly detrimental without any compensating benefit. The following, taken from the Savannah Morning News, shows the feeling in that city :

AN IMPORTANT MEASURE.

The following communication, received from the Secretary of the Board of Sanitary Commissioners, was read and adopted :

OFFICE BOARD SANITARY COMMISSIONERS,
SAVANNAH, March 29, 1880.

*To the Honorable Mayor and Aldermen City of Savannah :*

GENTLEMEN—I am instructed to transmit to your honorable body the following resolution, passed by the Board of Sanitary Commissioners at a meeting held this day :

*Resolved*, That this Board request the City Council of Savannah to direct His Honor the Mayor to communicate at once with our Senators and Representatives in Congress, and urge upon them a firm opposition to the passage of the health bills now before Congress to give additional powers to the National Board of Health, said bills being presented by Hons. J. G. Harris, C. Young and Jos. H. Acklen. This Board consider these bills prejudicial to the interests of our city. I am gentlemen, your obedient servant,

J. T. MCFARLAND, M.D.,
Secretary B. S. Commissioners.

In accordance with the above, the following resolutions were offered by Alderman Duncan :

WHEREAS, The bills now pending in Congress, increasing the powers of the National Board of Health, will, if enacted into laws, be prejudicial to the interests of our seaport cities, by imposing onerous restrictions upon our commerce. Therefore, be it

*Resolved* 1. That in the opinion of this Council, the bill proposed by Hon. Messrs. Harris and Young in Congress, entitled "A bill to increase the efficiency of the National Board of Health;" the bill introduced in the House by Mr. Acklen, entitled "A bill for the regulation of inter-State freight and passengers, and to relieve the same from the restrictions of local quarantines," and the bill introduced by Mr. King, to "increase the inefficiency of the National Board of Health," are intended to confer upon the National Board of Health powers far too great to be safely entrusted to any central body, inasmuch as they convey the *right to supersede* the authority of all State and local government in the matter of quarantine, and to *close any State* against intercourse with the rest of the world, whenever in the *opinion* of the National Board of Health or its Executive Committee (of five persons), an epidemic of infectious or contagious disease prevails in that State.

*Resolved* 2. That in the opinion of this Council, such legislation by the Federal Government is revolutionary in its tendencies, and contrary to the spirit of our institutions.

*Resolved* 3. That a copy of these resolutions be sent to the Hon. J. C. Nichols, Representative of the First District in Congress, with the request that he use his influence against the passage of the above bills.

*Resolved* 4. That His Honor the Mayor be requested to communicate with the State and municipal authorities, bordering on the Atlantic and Gulf coast, with a view to appointing delegates to a Convention, the object being the sanitary and commercial interests of the States, and the framing of such a bill, which, while looking to the health of the communities, will not compromise their commercial prosperity.

Alderman Duncan, for the information of those not posted in regard to the matter, stated the importance of action in the premises as the conferring of such powers upon the National Board of Health as provided in the bills now before Congress was dangerous in the extreme, and he plainly and forcibly set forth reasons why they should so be considered.

Alderman Blun did not favor the action, as he thought it would be misunderstood; did not think Savannah should be the first to move.

Alderman Duncan replied that this remark showed the Alderman not to be posted in regard to the matter, as other cities had already moved, and action was being taken all over the country.

The motion to adopt the recommendations of the Sanitary Board, and the resolutions of Alderman Duncan, was carried.

